# Cellular Senescence and Mammalian Healthspan

**DOI:** 10.1093/geroni/igaa057.2655

**Published:** 2020-12-16

**Authors:** Judith Campisi

**Affiliations:** The Buck Institute for Age Research, Novato, California, United States

## Abstract

Cellular senescence is a complex cell fate, often induced by stress or damage, that can be beneficial or deleterious, depending on the physiological context and age of the organism. A prominent feature of senescent cells is a multi-faceted senescence-associated secretory phenotype (SASP), which includes growth factors, cytokine and chemokines, growth factors, proteases, bioactive lipids and metabolites. Senescent cells increase with age in most, if not all, mammalian tissues. Through the use of transgenic mouse models, senescent cells are now known to causally drive numerous age-related pathologies, largely through the SASP. Eliminating senescent cells, genetically or through the use of senolytic/senomorphic agents, can improve the health span, at least in mice, and hold promise for extension to humans in the near future.

